# A 15 Mb large paracentric chromosome 21 inversion identified in Czech population through a pair of flanking duplications

**DOI:** 10.1186/1755-8166-7-51

**Published:** 2014-08-19

**Authors:** Jana Drabova, Marie Trkova, Miroslava Hancarova, Drahuse Novotna, Michaela Hejtmankova, Marketa Havlovicova, Zdenek Sedlacek

**Affiliations:** 1 Department of Biology and Medical Genetics, Charles University 2nd Faculty of Medicine and University Hospital Motol, Prague, Czech Republic; 2 Gennet, Prague, Czech Republic

**Keywords:** Inversion, Duplication, *USP25*, SNP arrays, Founder effect

## Abstract

**Background:**

Inversions are balanced structural chromosome rearrangements, which can influence gene expression and the risk of unbalanced chromosome constitution in offspring. Many examples of inversion polymorphisms exist in human, affecting both heterochromatic regions and euchromatin.

**Results:**

We describe a novel, 15 Mb long paracentric inversion, inv(21)(q21.1q22.11), affecting more than a third of human 21q. Despite of its length, the inversion cannot be detected using karyotyping due to similar band patterns on the normal and inverted chromosomes, and is therefore likely to escape attention. Its identification was aided by the repeated observation of the same pair of 150 kb long duplications present in cis on chromosome 21 in three Czech families subjected to microarray analysis. The finding prompted us to hypothesise that this co-occurrence of two remote duplications could be associated with an inversion of the intervening segment, and this speculation turned out to be right. The inversion was confirmed in a series of FISH experiments which also showed that the second copy of each of the duplications was always located at the opposite end of the inversion. The presence of the same pair of duplications in additional individuals reported in public databases indicates that the inversion may also be present in other populations. Three out of the total of about 4000 chromosomes 21 examined in our sample carried the duplications and were inverted, corresponding to carrier frequency of about 1/660. Although the breakpoints affect protein-coding genes, the occurrence of the inversion in normal parents and siblings of our patients and the occurrence of the duplications in unaffected controls in databases indicate that this rare variant is rather non-pathogenic. The inverted segment carried an identical shared haplotype in the three families studied. The haplotypes, however, diverged very rapidly in the flanking regions, possibly pointing to an ancient founder event at the origin of the inversion.

**Conclusions:**

The identification of inv(21)(q21.1q22.11) supports the notion that paracentric inversions are the most common form of chromosomal variation and that some of them may still remain undetected.

## Background

Since the identification of the cytogenetically visible heterochromatic inversion inv(9)(p11q12) almost half a century ago [1], many other polymorphic inversions involving both heterochromatin (1qh, 16qh and Yqh) and euchromatin have been described in human [2]. The most common euchromatic inversions are the 22–25 Mb long inv(2)(p11.2q13), the 22.6 Mb long inv(10)(p11.2q21.2) and the very frequent 4.7 Mb long inv(8)(p23.1) [3-7]. Paired-end sequencing and other focused approaches have recently led to the identification of many smaller inversions. The Database of Genomic Variants currently lists 583 merged inversion regions, and the InvFEST database lists 85 well validated human inversions [8,9].

Inversions often have no clinical significance, although opposite examples exist, like the recurrent inversion in the *F8* gene causing haemophilia A [10]. Therefore the identification of a rare inversion prenatally or in an affected child always possesses a question of a possible phenotypic influence. Inversion carriers also have a risk of unbalanced offspring; considering paracentric and pericentric inversions together, the risk is about 1% [7]. Two mechanisms contribute to the formation of unbalanced gametes in inversion carriers. First, an odd number of recombinations within the inversion loop in an inversion heterozygote may lead to aberrant chromosomes (with a higher risk in pericentric inversion heterozygotes producing duplication-deletion chromosomes and a lower risk in paracentric inversion heterozygotes producing dicentric and acentric recombinant chromosomes expected to be lethal) [11,12]. Second, due to a less favourable arrangement of low-copy repeats at the breakpoints of the inverted allele, several inversions have been described to predispose to rearrangements including those associated with some microdeletion syndromes [13,14].

Two scenarios exist for the evolutionary origin of human polymorphic inversions: they can be either recurrent, or can descend from a single ancestral event. While the inv(2)(p11.2q13) is recurrent due to the local genome architecture [6], the inv(10)(p11.2q21.2) seems to have just one ancestral founder in Northern Europe [5], and similarly the inv(8)(p23.1) shows a strong founder effect [3]. A large study of different inversions concluded that recurrence is rather exceptional and that most inversions are likely to be identical by descent [15].

In this report we describe a serendipitous identification of inv(21)(q21.1q22.11), a large, 15 Mb long paracentric inversion affecting more than a third of the long arm of human chromosome 21 (chr21), which cannot, however, be identified using karyotyping. Repeated identification of the same pair of small duplications in cis on chr21 in three Czech families using single nucleotide polymorphism (SNP) arrays prompted us to hypothesise that this remarkable co-occurrence of two remote copy number variants (CNVs) could be specific for an unusual rare structural variant of chr21, for example one with an inversion of the DNA segment flanked by the duplications, and this scenario was confirmed by multiple experiments. The inversion may not be restricted to the three families reported, as suggested by the presence of the same pair of duplications in additional individuals reported in public CNV databases.

## Results

**Figure 1:**
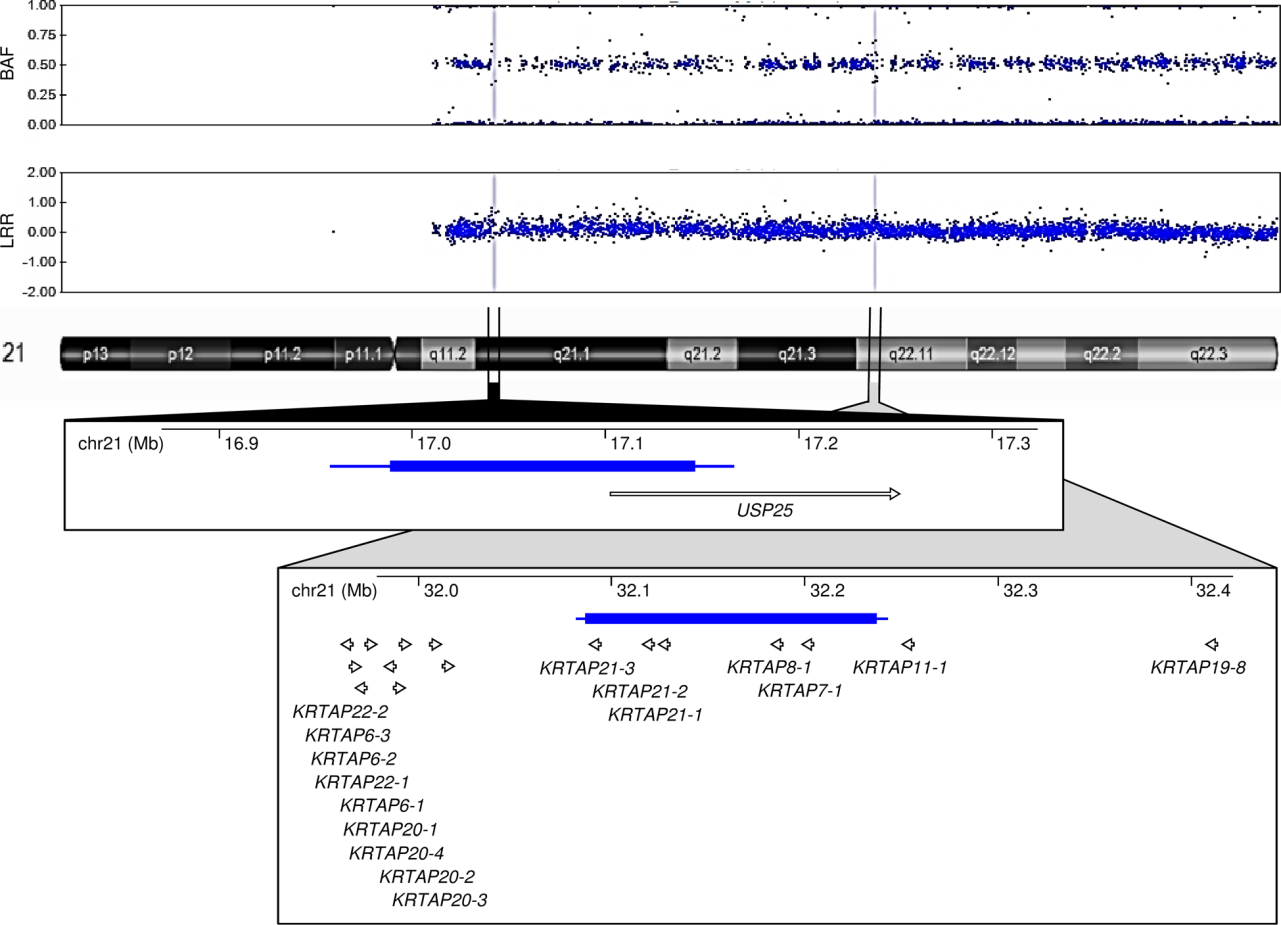
**An example of the SNP array result in a chr21 duplication carrier and gene content of the two concurrent chr21 duplications.** LRR, log R ratio; BAF, B allele frequency. Thick and thin blue bars in the schematics represent the minimum and maximum span of the duplications, respectively. Vertical double lines mark the regions of the duplications. Open arrows show the position and orientation of genes. Chromosome 21 megabase scale is also added.

Families A-C were referred for genetic testing because of intellectual disability (ID), autism and other phenotypes in their children. Karyotyping of the family members showed apparently normal results. SNP array analysis identified similar pairs of duplications on chr21 of about 150 kb each located in a distance of about 15 Mb initially in the affected children from Families A and B and in an unaffected sibling from Family C. Later the same pairs of duplications were also identified in an unaffected father from Family A and in unaffected mothers from Families B and C. Automated analysis followed by manual inspection showed that the breakpoint intervals of both duplications were identical in all six carriers (arr 21q21.1(16,957,141×2, 16,988,490-17,146,328×3, 17,166,671×2) and arr 21q22.11(32,080,968×2, 32,086,323-32,236,820×3, 32,242,774×2)). The proximal duplication involved the 5' region of the *USP25* gene including three to five of its 5' exons. The distal duplication was located in the *KRTAP* cluster and contained five members of this gene family (*KRTAP21-3*, *KRTAP21-2*, *KRTAP21-1*, *KRTAP8-1* and *KRTAP7-1*) (Figure 1). The breakpoint intervals did not contain segmental duplications or any remarkable enrichment for dispersed repeats. With the exception of common polymorphisms, no additional significant CNVs were found in the individuals tested from Families A-C.

**Figure 2:**
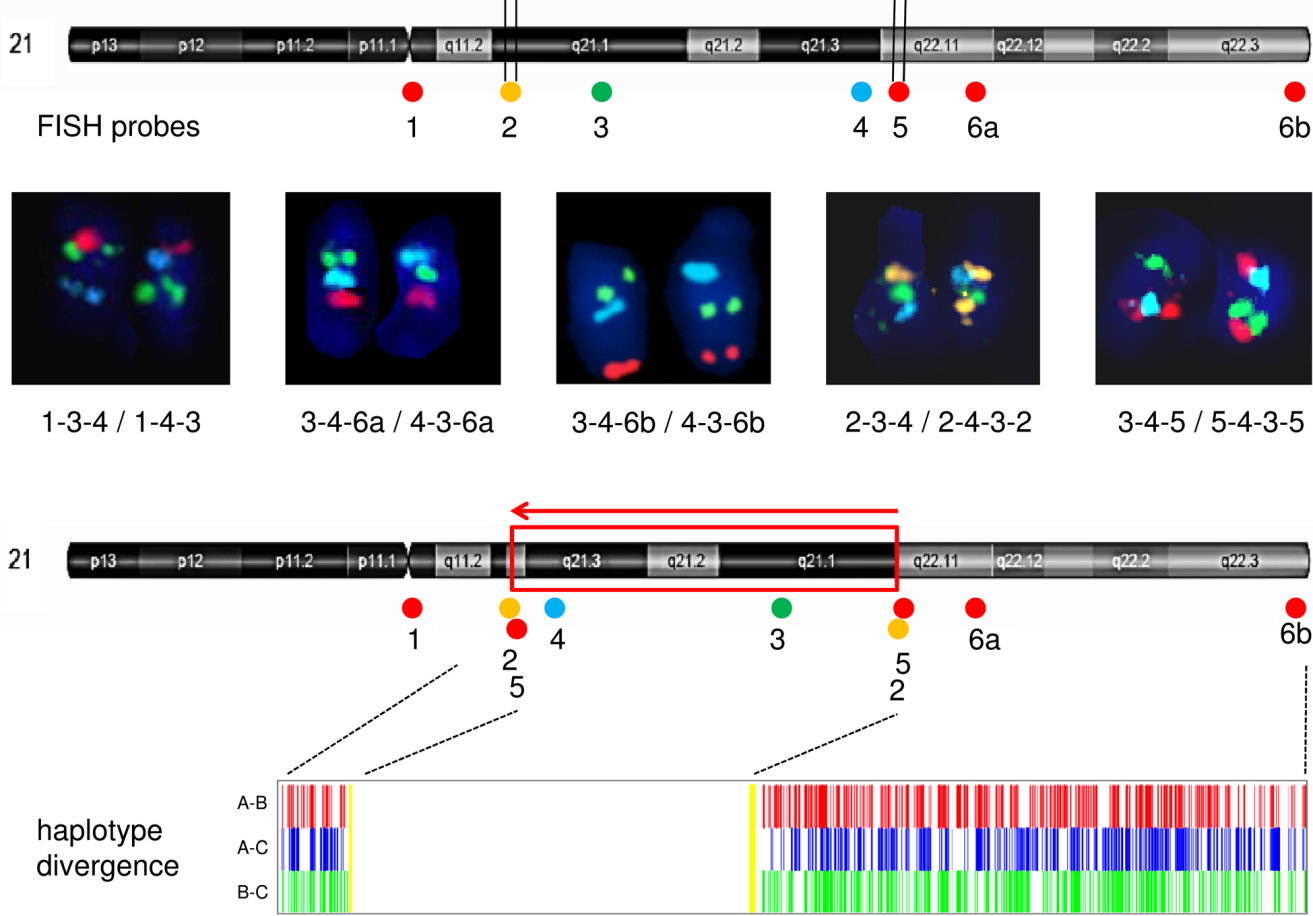
**Location of FISH probes used in the study, results of FISH experiments, and structure of the chr21 with inv(21)(q21.1q22.11) and its haplotypes.** The numbering of FISH probes corresponds to Table 1. The colours of the dots used to mark the positions of the FISH probes reflect the colours of fluorochromes used. Vertical double lines mark the regions of the duplications. The hybridisation of various combinations of FISH probes on the normal (left) and inverted (right) chr21 is shown. The bottom chromosome ideogram shows the inverted chromosome (with the inversion boxed in red and marked with a red arrow) including the new positions of the FISH probes. The schematics at the bottom of the figure shows haplotype divergence with genotypes discordant among Families A-B, A-C and B-C indicated in red, blue and green, respectively, and the duplications flanking the insertion in yellow. Note the absolute haplotype identity (no discordant genotypes) within the inversion.

The occurrence of identical pairs of chr21 duplications in the children and their parents implicated that the duplications were always in cis on one chr21. This unusual repeated co-occurrence of two aberrations on one chr21 prompted us to hypothesise that the DNA segment flanked by the duplications might be inverted on these chr21. The inversion status was tested using fluorescence in situ hybridisation (FISH). Using two probes internal and close to opposite ends of the segment flanked by the duplications combined with a probe located either centromeric or telomeric (probe combinations 1-3-4 and 3-4-6a/6b, Table 1), we showed that this segment was indeed inverted on one chr21 in the duplication carriers, and confirmed that the inversion was paracentric (Figure 2). In addition, using probes located within both duplications (probe combinations 2-3-4 and 3-4-5, Table 1) we showed that the second copy of each of the duplications was always located at the opposite end of the inverted segment, i.e. that the duplications were not tandem but rather insertional and flanking the inversion (Figure 2). The relative arrangement of the duplicated copies at the ends of the inverted segment could not be studied on metaphase chr21. However, the majority of interphase nuclei studied using probe combination 2-3-4-5 showed signals ordered 2-5-4-3-2-5 on the inverted chr21, and long-range PCR experiments indicated that the two copies of the distal duplicated segment on the inverted chr21 were adjacent to their normal flanking sequence just at one of their two ends (data not shown). This would be consistent with a hypothetic scenario in which the whole segment delineated by probes 2-3-4-5 was used in its inverted orientation to replace the segment 3–4 on the derivative chr21, with the flanking segments (2 and 5) preserved in their original positions.The rearrangement was identified on three unrelated chr21 out of a total of 3936 chr21 analysed, giving approximate allele frequency of 0.0008 and carrier frequency of 0.0015 (~1/660). The availability of SNP genotypes in the three family trios (two parents - child trios and one mother - two half sibs trio) with two inversion carriers in each trio allowed us to deduce the haplotypes of the inversion chr21. Out of 1701 markers present in the inverted segment (including the duplications), in 1386 markers the SNP allele was identical in all three families, and in none of the remaining markers the genotypes were at odds with a common shared haplotype (in 252 markers two families shared the same allele and in the third family data were not available or the family was not informative; in the remaining 63 markers data were available just from one or from none of the families). Therefore the haplotype within the inverted segment was likely to be identical on all three inverted chr21 studied. However, outside of the inversion the haplotypes diverged rapidly: the haplotype in Family B diverged already 66 kb proximally and 229 kb distally from the inversion, and haplotypes in the remaining two families mutually diverged 330 kb proximally and 490 kb distally from the inversion (Figure 2).

**Table 1:** FISH probes used in the study

**No.**	**Probe description**	**Probe specification (Supplier)**	**Colour**
1	chr21 centromeric probe (proximal to both duplications)	D21Z1 (Cytocell, Cambridge, UK)	red
2	probe overlapping the proximal duplication	RP11-482O14 (BlueGnome, Cambridge, UK)	orange
3	probe at the proximal end of the segment between the duplications	RP11-45 M19 (Source BioScience, Nottingham, UK)*	green
4	probe at the distal end of the segment between the duplications	RP11-64I20 (Source BioScience)*	blue
5	probe overlapping the distal duplication	RP11-134 K3 (Source BioScience)*	red
6a (6b)	DSCR1 (or chr21qter subtelomere) probe (distal to both duplications)	PN21 (Kreatech Diagnostics, Amsterdam, The Netherlands) (or D21S1575 (Cytocell))	red

*Human Genomic Clone Set V1.0.

## Discussion

We identified a pair of identical duplications on three out of a total of about 4000 unrelated human chr21 tested, and we showed that the 15 Mb segment flanked by the duplications forms a yet unknown large paracentric inversion of chr21. A thorough database and literature search revealed the same combination of similar duplications in one patient with developmental delay (individual 9885009, chr21:16,956,885-17,141,961 (nssv1172163) and chr21:32,098,070-32,217,892 (nssv1172207)) and in one unaffected control (individual 1780854261_A, chr21:16,956,866-17,145,011 (nssv1151408) and chr21:32,086,323-32,224,934 (nssv1151611)) from one study [16], and in one unaffected control (individual SW_0033, chr21:16,948,663-17,145,770 (essv7004290) and chr21:32,082,962-32,242,849 (essv7004391)) from another study [17]. Several other individuals were reported to carry just one of the duplications; taking into account the relatively small size of the segments, it is possible that some array platforms or CNV calling algorithms could overlook one duplication from the pair. By analogy with our results, it could be expected that these chromosomes also carry the inversion. This together with our estimate of carrier frequency of about 1/660 may indicate that the inversion could be more common, and that it may not be limited to the Czech population.

This notion can be further supported by the observation of chr21 haplotypes rapidly diverging outside of the inverted region, confirming that the Czech inversion carriers studied were not closely related. At the same time the shared identical haplotype within the inverted segment pointed to the common descent of the three inverted chr21 from a single ancestral event, a scenario observed in most inversions [15]. Although the very large size of the inversion may have allowed for double crossing over, thus limiting linkage disequilibrium only to regions adjacent to the breakpoints of the inverted segment, the haplotypes observed in our families were strictly uniform throughout the whole segment. This may support the view that recombination is actively suppressed in inverted regions [18,19]. The inverted segment contains hot spots of variable recombination rate which can potentially contribute to this phenomenon [20].

The inheritance of the rearranged chr21 from healthy parents in Families A-C and its presence in an unaffected child from Family C together with the likely occurrence of the inversion in unaffected controls from public databases indicate that this rearrangement is unlikely to be a rare pathogenic variant responsible for the abnormal phenotype in patients from our Families A and B or in individual 9885009 [16]. The repeated identification of the inversion in affected persons may simply reflect the proportion of affected patients tested using microarrays among all individuals analysed, at least in the Czech cohorts. However, it cannot be excluded that this rare variant could represent a predisposing factor with incomplete penetrance and/or variable expressivity possibly contributing to neurodevelopmental disorders in conjunction with other genetic, epigenetic or environmental factors.

The proximal duplication involves *USP25*, a ubiquitously expressed gene coding ubiquitin specific peptidase 25, a protease which breaks polyubiquitin chains on degraded proteins and is thus involved in ubiquitin metabolism and recycling. Defects in other ubiquitin specific peptidases, for example *USP15*, have been associated with autism [21]. It is unclear if the rearranged chr21 carries one intact copy of *USP25*, and if the expression of this copy is influenced by a possible positional effect associated with the rearrangement. The distal duplication harbours five members of the *KRTAP* gene family coding keratin associated proteins present in the interfilamentous matrix of the hair cortex. The five small genes are fully embedded in the duplication and the influence of the rearrangement on their expression or the expression of other neighbouring members of the *KRTAP* family is also unclear. In addition to genes directly affected by the breakpoints or by the duplications, other genes from the inversion or its flanking regions can be influenced, as described in other inversions [22].

There was no remarkable history of infertility, spontaneous abortions or developmental defects in extended pedigrees of inv(21)(q21.1q22.11) carrier parents from Families A-C (multiple abortions in Family A were reported in the maternal branch which, however, did not carry the inversion). This is in accord with the generally expected low risk of abortions in inversion carriers or of children carrying recombinant chromosomes [7], which can in turn be associated with the suppression of recombination within the inverted segment [18,19]. Nevertheless, evidence exists that some types of recombination (the U-type exchange within the inversion loop) or the breakage of dicentric recombinant chromosomes can produce abnormal gametes capable of giving rise to affected offspring in paracentric inversion carriers [4,23]. The literature search has not revealed any inv dup del(21q) which could be associated with inv(21)(q21.1q22.11); however, due to the position of the inversion, the inv dup del arrangement would have a very large terminal deletion of 21q and its carriership may not be compatible with life. A patient has been described with a combination of symptoms of proximal 21q monosomy and some symptoms of Down syndrome who carried an analphoid marker chr21 consisting of inverted duplication of 21q21.1qter with a neocentromere [24]. A similar marker chr21 could originate as an acentric recombination product in an inv(21)(q21.1q22.11) heterozygote; however, identical microsatellite alleles on both segments pointed in this case to an intrachromosomal origin of the rearrangement, or to an additional recombination immediately distal to the distal inversion breakpoint [24]. Another case with a *de novo* 9-break complex chr21 rearrangement had breakpoints in band 21q22.11 [25]; however, the resolution of the study did not allow deciding if the breakpoints were identical with those on the inverted chr21 described here.

Although the inversion affects 15 Mb of DNA and more than a third of the long arm of chr21, due to no observable difference in the banding pattern of the inverted chr21 this rearrangement is likely to escape attention during karyotyping. There are several reports in the literature of rare paracentric inversions in 21q identified cytogenetically [reviewed in [26], but they seem to be different, and their identification in the light microscope in fact rather argues against them being exactly identical with the current inversion.

## Conclusions

Our identification of the inv(21)(q21.1q22.11) together with another example of a recent discovery of a large inv(10)(q11.22q21.1) [27] can indicate that paracentric inversions may indeed be the most common form of chromosomal variation, of which some examples still remain undetected [12].

## Methods

### Cohorts

The research was in compliance with the Helsinki Declaration and was approved by the local ethics committee (University Hospital Motol) under reference number EK 597/09. Informed consent with participation in the study was obtained from all subjects or their legal guardians.

SNP array analysis was performed in three Czech patient cohorts. The first cohort consisted predominantly of children with a broad range of phenotypes including developmental delay, ID, autism, seizures, and congenital and growth anomalies, and involved 1298 unrelated patients representing 2596 chr21. In 88 of these patients both parents and in 33 patients one parent were also analysed on the array, thus adding 209 unrelated chr21 and totalling the number of chr21 in this cohort to 2805. The second cohort consisted of 477 foetuses referred for prenatal SNP array testing, with both parents or one parent analysed in 27 or 11 cases, respectively, totalling the number of chr21 to 1019. The third cohort consisted of 28 couples analysed as a part of pre-conception care, who harboured 112 chr21. The three cohorts thus yielded a grand total of 3936 chr21.

### Families

Family A was ascertained through a female patient with moderate ID and hyperactivity. At the age of 9.5 years she had a long face, frontal bossing, long nose with a broad tip and large nares, epicanthal folds, upslanted palpebral fissures, big open mouth with a narrow upper lip and prominent lower lip, sparse teeth and small low-set dysplastic protruding ears. Her face resembled that of patients with Angelman syndrome, but testing for this condition was negative. She also showed wide stance with bent knees, long fingers on both hands and feet, and pedes planovalgi. Her growth was normal. The patient had an older step brother and two younger brothers who, however, were not available for analysis. The youngest brother suffered from developmental delay and hyperactivity. Both parents of the patient finished primary education. The mother had a history of one spontaneous abortion and there were multiple occurrences of abortions in the maternal branch of the family. SNP array testing was performed in the patient, and later also the parents were analysed.

Family B was ascertained through a currently 13-year-old boy with moderate to severe ID and autism with no language (he used only three words). The boy showed attacks of anger, aggression and self-aggression. His facial features included a long face, frontal bossing, distinct arched eyebrows, upslanted palpebral fissures, depressed nasal bridge, upturned nasal tip, big mouth with a thin upper lip, prominent incisors and small low-set ears. He also showed poor posture, wide stance with bent knees, genua valga and macrogenitalism. His mother was a high school graduate and worked as a librarian. His father was trained as a motor mechanic. The boy had one healthy older brother. Family B had no history of infertility or spontaneous abortions. A paternal aunt of the father suffered from epilepsy. Other genealogical data were unremarkable. Microarray analysis was performed in the patient and his parents.

Family C was identified through a currently 13-year-old boy with autism, unsteady gait and symptoms suggestive of neurofibromatosis including eye symptoms and cafe-au-lait spots. Parallel to the microarray analysis, a probably pathogenic missense *NF1* mutation was identified in the patient (*de novo* or paternal, the father was not available). The patient had two apparently healthy siblings, a 15-year-old brother and a 3-year-old step brother. The family history was unremarkable and there was no history of infertility or spontaneous abortions. The family insisted on microarray analysis of the patient and his younger step brother. Subsequently also the mother of both boys was analysed.

### Laboratory methods

Karyotyping of peripheral blood lymphocytes was performed using standard methods. Genomic DNA of individuals from Families A-C was tested using Human CytoSNP-12 BeadChips (~300 K; Illumina, San Diego, CA, USA). Array data were analysed using GenomeStudio (Illumina) and QuantiSNP [28]. FISH employed standard protocols and various combinations of probes listed in Table 1. The study used genome build hg19.

## Consent

Written informed consent was obtained from the patients' parents for the publication of this report and any accompanying images.

## Abbreviations

chr21: Chromosome 21; CNV: Copy number variant; FISH: Fluorescence in situ hybridisation; ID: Intellectual disability; SNP: Single nucleotide polymorphism.

## Competing interests

The authors declare that they have no competing interests.

## Authors’ contributions

MT and MHan carried out the microarray and molecular genetic studies. JD, DN and MT carried out karyotyping and the FISH experiments. MHav and MHe performed the clinical genetics part of the study. MHan, MT and ZS analysed the haplotypes. ZS, MT, JD and MHan conceived the study, participated in its design and coordination, analysed the data and drafted the manuscript. All authors have read and approved the final manuscript.
